# CD44 is involved in liver regeneration through enhanced uptake of extracellular cystine

**DOI:** 10.1002/ctm2.873

**Published:** 2022-05-11

**Authors:** Hyun Young Kim, Ga Hee Baek, Wonseok Lee, Young Joo Lee, Wan Seob Shim, Young Jae Choi, Byung‐Hoon Lee, Sang Kyum Kim, Keon Wook Kang

**Affiliations:** ^1^ College of Pharmacy and Research Institute of Pharmaceutical Sciences Seoul National University Seoul Republic of Korea; ^2^ College of Pharmacy Chungnam National University Daejeon Republic of Korea

Dear Editor,

Liver regeneration triggered by fulminant liver damage accompanies the proliferation of hepatic progenitor cells (HPCs). Thus, there has been considerable interest in identifying specific HPC markers. However, the physiological role of HPC markers during liver regeneration is not fully understood. Herein, we identify the mechanistic roles of CD44, a cell surface marker for hepatic regeneration,^1^ in HPC proliferation and redox homeostasis.

The Gene Expression Omnibus data revealed an increase in CD44 expression after partial hepatectomy (Figure 1A). Remnants of the mouse left hepatic lobe, which had undergone resection of more than 80% of its volume, showed significant elevation of CD44 expression. CD44 expression peaked at 10 days after the resection, at which time, the level of the proliferation marker, proliferating cell nuclear antigen (PCNA), was the highest (Figure 1B, Figure S1A). We isolated primary hepatocytes from the liver 10 days after resection and confirmed the increase of CD44 expression in the hepatocytes (Figure 1C–E). Acetaminophen (APAP)‐induced acute liver injury also increased hepatic CD44 expression.^2^ The expression of CD44 peaked at 48 h after APAP treatment (Figure 1F). Primary hepatocytes from mice after APAP injection showed higher levels of PCNA and CD44 than did hepatocytes from phosphate‐buffered saline (PBS)‐injected mice (Figure 1G,H).

**FIGURE 1:**
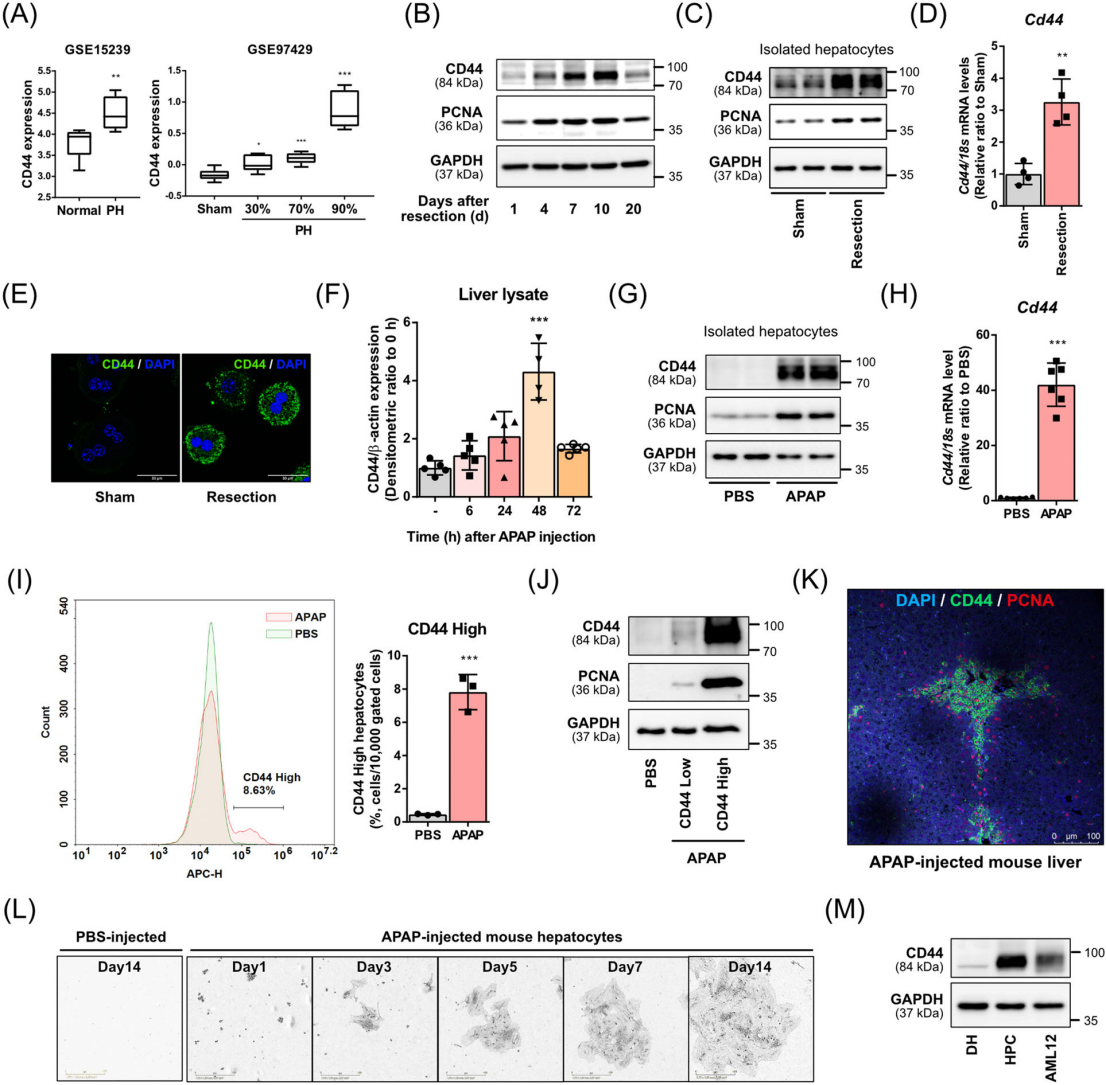
Expression of CD44 is up‐regulated in regenerative hepatocytes. (A) CD44 expression in liver specimens from patients shortly (.5 h, 1 h and 1.5 h) after partial hepatectomy (GSE15239). CD44 expression in liver samples from rats 24 h after partial hepatectomy (PH) was analysed according to the size of the withdrawn livers (GSE97429). (B) Mice were sacrificed after the indicated day following liver resection. Tissue lysates from the remnant left lobe were analysed by immunoblot analysis. (C) Primary hepatocytes were isolated from mice 10 days after liver resection. Protein levels of proliferating cell nuclear antigen (PCNA) and CD44 were measured using immunoblot analyses. (D) Messenger RNA (mRNA) level of *Cd44* was quantified using quantitative polymerase chain reaction (qPCR). *n* = 4 per group. (E) Immunofluorescence images (scale bars, 30 µm) of CD44 in primary hepatocytes from mice 10 days after liver resection. Representative dataset from *n* = 3 per group. (F) Mice were sacrificed at the indicated time points following 250 mg/kg APAP injection. *n* = 4 or 5 mice per group. ***p < .001 compared to the PBS group, analysed by one‐way analysis of variance (ANOVA) followed by Dunnett's test. (G and H) Primary hepatocytes were isolated from mice 48 h after APAP injection. Protein levels of PCNA and CD44 and mRNA level of *Cd44* were quantified in the isolated primary hepatocytes from APAP‐injected mice *n* = 6 per group. (I) Flow cytometry analysis of CD44‐APC in hepatocytes isolated from APAP‐injected mice. *n* = 3 mice per group. APC, allophycocyanin. (J) CD44‐expressing hepatocyte population was sorted using cell sorter and lysed to evaluate the expression level of PCNA. (K) Immunofluorescence images (scale bar, 100 µm) of CD44 and PCNA in liver tissues from mice 48 h after APAP injection. Representative dataset from *n* = 3 per group. (L) Isolated small hepatocytes from PBS or APAP‐injected mice were seeded on a hyaluronic acid (HA)‐coated dish. Representative images (scale bars, 400 µm) of cultured cells were captured on the indicated day using IncuCyte S3. (M) CD44 levels were quantified in differentiated hepatocytes (DH), hepatic progenitor cells (HPCs), and AML12 cells. (A, D, H and I) Data are presented as mean ± standard deviation (SD); *p < .05, **p < .01 and ***p < .001 compared to normal, sham or PBS group, analysed by unpaired student's *t*‐test

Approximately 8% of the hepatocytes from APAP‐injected mice constituted a discrete population with a high CD44 level (Figure 1I). The subpopulation of hepatocytes also highly expressed PCNA and an HPC marker,^3^ EpCAM (Figure 1J,K, Figure S1B). To further identify the CD44‐expressing hepatocytes as HPCs, we verified their proliferation capacity. Considering the role of CD44 as an adhesion molecule that binds to hyaluronic acid (HA), we isolated small hepatocytes (Figure S1C) and cultured them on an HA‐coated dish.^4^ The small hepatocytes isolated from APAP‐injected mice proliferated and formed colonies (Figure 1L). The cultured hepatocytes highly expressed CD44 with a low level of albumin, a representative marker for differentiated hepatocyte (Figure S1D). Furthermore, AML12, a murine hepatocyte cell line, expressed a high level of CD44 and also proliferated on the HA‐coated dish, indicating that AML12 cells could be a surrogate cell line for HPCs (Figure 1M, Figure S1E).

To assess the role of CD44 in proliferative hepatocytes, we knocked down CD44 in AML12 (Figure 2A). CD44 knockdown significantly enhanced *tert*‐butyl hydrogen peroxide‐induced reactive oxygen species (ROS) generation (Figure 2B). Moreover, the intracellular glutathione (GSH) level was diminished by CD44 knockdown (Figure 2C). Isotope tracing using universal ^13^C_6_‐cystine revealed that more than 90% of the intracellular GSH was derived from extracellular cystine (M3) in AML12 (Figure S2A). System x_c_^−^ is a major antiporter that transports extracellular cystine for intracellular glutamate.^5^ The expression of xCT was significantly decreased after knockdown of CD44 (Figure 2D,E, Figure S2B,C). The activity of the system x_c_^−^ antiporter was measured using an extracellular glutamate assay and universal ^13^C_6_‐cystine isotope tracing. Knockdown of CD44 hindered the activity of system x_c_^−^ (Figure 2F,G).

**FIGURE 2:**
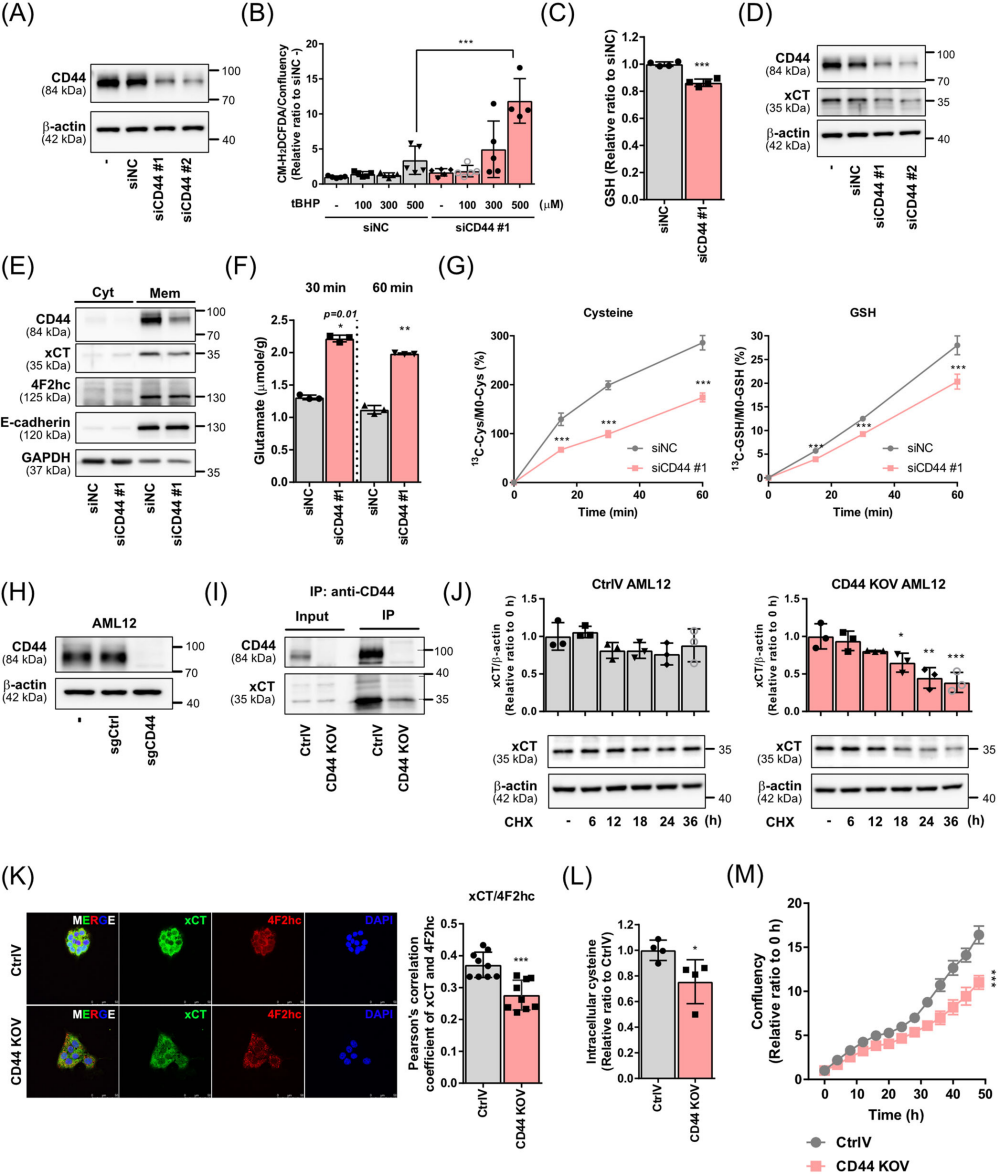
Silencing of CD44 inhibits system x_c_^−^ activity. (A) CD44 was knocked down in AML12 using siRNA. ‐, non‐transfected AML12; siNC, AML12 transfected with negative control siRNA; siCD44 #1 or siCD44 #2, AML12 transfected with siCD44#1 or #2. (B) ROS generation was measured using chloromethyl‐H_2_DCFDA after incubation with tertiary‐butyl hydroperoxide (tBHP) in siNC‐ or siCD44‐transfected AML12. *n* = 4 per group; ***p < .001 compared to the indicated group, analysed by one‐way ANOVA followed by Tukey's test. (C) Intracellular GSH level was measured in CD44‐knockdown AML12. (D) Protein expression of xCT was measured in CD44‐knockdown AML12. (E) AML12 cells transfected with CD44 siRNA were fractionated to obtain plasma membrane and cytosol samples and then subjected to immunoblot analysis. E‐cadherin and glyceraldehyde 3‐phosphate dehydrogenase (GAPDH) were assessed as protein markers for plasma membrane and cytosol fraction, respectively. Cyt, cytosolic fraction; Mem, membrane fraction. (F) Extracellular glutamate was determined in culture media 30 or 60 min after incubation. (G) siNC‐ or siCD44#1‐transfected AML12 cells were incubated with media containing universal ^13^C_6_‐cystine. Isotope‐labeled cysteine and isotope‐labeled GSH were measured in cell lysates after the indicated time. (H) Stable CD44‐knockout AML12 cells were produced using CRISPR‐Cas9. (I) Lysates of Crispr‐sgCtrl (CtrlV) and Crispr‐sgCD44 (CD44 KOV) cells were subjected to immunoprecipitation using CD44 antibody. (J) AML12 CtrlV or CD44 KOV cells were incubated with 40 µg/ml cycloheximide for indicated times. Immunoblot analysis of xCT and β‐actin (loading control) and the densitometric ratio of xCT/β‐actin band intensity relative to the corresponding value for the zero‐time point (0). Data are presented as mean ± SD; *p < .05, **p < .01 and ***p < .001 compared to the value of 0 h, analysed by one‐way ANOVA followed by Dunnett's test. (K) Immunofluorescence analysis of xCT and 4F2hc in AML12 CtrlV or CD44 KOV cells (scale bars, 50 µm). The colocalization between xCT and 4F2hc was quantified using Pearson's correlation coefficient (three pictures per sample, *n* = 3 per group). (L) Relative ratios of intracellular cysteine in AML12 CtrlV or CD44 KOV cells were quantified by liquid chromatography‐tandem mass spectrometry (LC‐MS/MS). *n* = 4 per group. (M) Proliferation rates of CtrlV or CD44 KOV‐expressing cells were measured using IncuCyte S3. *n* = 5 per group. (C, F and K–M) Data are presented as mean ± SD; *p < .05, **p < .01 and ***p < .001 compared to the value of siNC or CtrlV, analysed by unpaired student's *t*‐test

We established stable CD44‐knockout AML12 (Figure 2H). Immunoprecipitation analysis revealed that CD44 binds to xCT (Figure 2I, Figure S2D). Sulforaphane‐induced xCT expression was diminished, but basal expression of xCT was not significantly changed after CD44 deletion (Figure S2E). In fact, stable knockout of CD44 increased transcription of xCT (*Slc7a11*) and 4F2hc (*Slc3a2*) (Figure S2F). We confirmed that nuclear factor erythroid 2‐related factor 2, a major transcription factor of xCT,^6^ was activated in the CD44‐knockout AML12 (Figure S2G–I). We estimate that the oxidative stress‐responsive signaling blunted the effect of CD44 on system x_c_^−^.

Meanwhile, CD44 ablation facilitated the protein degradation of xCT (Figure 2J). The decreased protein stability impeded formation of the 4F2hc/xCT complex (Figure 2K). Deletion of CD44 significantly inhibited glutamate excretion (Figure S2J) and decreased the intracellular levels of cysteine and GSH (Figure 2L, Figure S2K). We also found that the limitations in cysteine and GSH resulted in a marked decrease in the proliferation rate (Figure 2M). In addition to system x_c_^−^‐mediated cystine uptake, cysteine can also be synthesized from methionine through the transsulfuration pathway composed of cystathionine β‐synthase and cystathionine γ‐lyase (CSE).^7^ Expression of CSE was significantly increased after CD44 deletion. However, ablation of CD44 showed neither increase nor decrease of transsulfuration enzyme activity (Figure S2L,M).

Primary hepatocytes were isolated from mice 48 h after APAP injection. Although the transcript levels of xCT did not change in the isolated primary hepatocytes (Figure 3A), APAP injection up‐regulated the expression of xCT in the plasma membrane (Figure 3B). The activity of system x_c_^−^ was markedly up‐regulated by APAP injection (Figure 3C–F, Figure S3A). The functional involvement of system x_c_^−^ in proliferative hepatocytes was assessed using erastin, a selective inhibitor of system x_c_^−^. Although differentiated hepatocytes were resistant to erastin treatment, HPCs and AML12 were highly sensitive to it (Figure 3G, Figure S3B). Overall, the data revealed that system x_c_^−^ is necessary for HPC proliferation.

**FIGURE 3:**
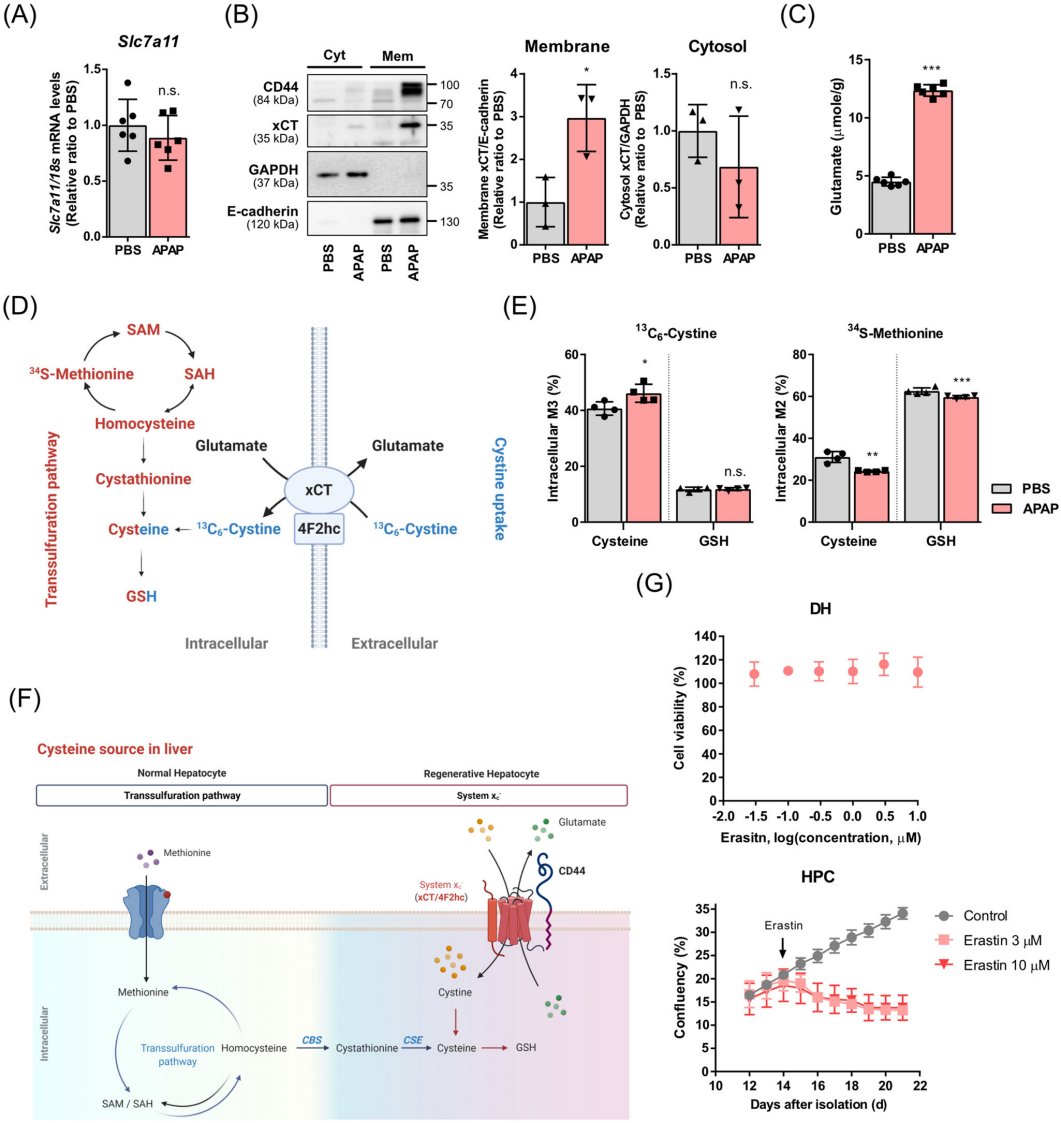
APAP‐induced xCT expression in proliferative hepatocytes increases cystine uptake. (A) Primary hepatocytes were isolated from mice 48 h after PBS or APAP injection. xCT (*Slc7a11*) mRNA levels of the hepatocytes were measured using qPCR. (B) The hepatocytes were fractioned to obtain plasma membranes and cytosol samples and then used for immunoblot analysis. The levels of E‐cadherin and GAPDH were assessed as protein markers for plasma membrane and cytosolic fraction, respectively. Cyt, cytosolic fraction; Mem, membrane fraction. (C) Exported glutamate level was quantified in the media after incubation of the hepatocytes for 5 h. (D) Graphical representation of transsulfuration pathway and system x_c_^−^‐mediated glutathione synthesis. (E) Isolated primary hepatocytes from mice 48 h after PBS or APAP injection were incubated in cystine‐free medium supplemented with universal ^13^C_6_‐labeled cystine or methionine‐free medium supplemented with ^34^S‐labeled methionine for 6 h. The bar graph shows the proportions of labeled‐ metabolites in cell lysates. (F) Proposed graphical representation of system x_c_^−^‐mediated glutathione synthesis in hepatic progenitor cell. (G) Relative cell viability of differentiated hepatocyte (DH) upon treatment with erastin for 48 h. The effect of erastin on cell proliferation of hepatic progenitor cell (HPC) was assessed using IncuCyte S3. (A–C and E) Data are presented as mean ± SD; *p < .05, **p < .01, ***p < .001 and n.s. not significant, compared PBS‐injected group, analysed by unpaired student's *t*‐test

We addressed the issue of whether system x_c_^−^‐dependent cystine uptake plays a critical role in liver regeneration. To that end, C57BL/6 mice were administered a metabolically stable system x_c_^−^ inhibitor, imidazole ketone erastin^8^ (IKE), 24 h immediately following APAP injection (Figure 4A). IKE administration significantly abrogated the increase of proliferation markers in APAP‐injected mice without increasing liver damage at later time points (Figure 4B–D, Figure S4A–D). IKE administration retarded the increase of HPC markers (Figure 4E). These results indicate that HPC‐mediated cell proliferation was inhibited by blockade of system x_c_^−^.

**FIGURE 4:**
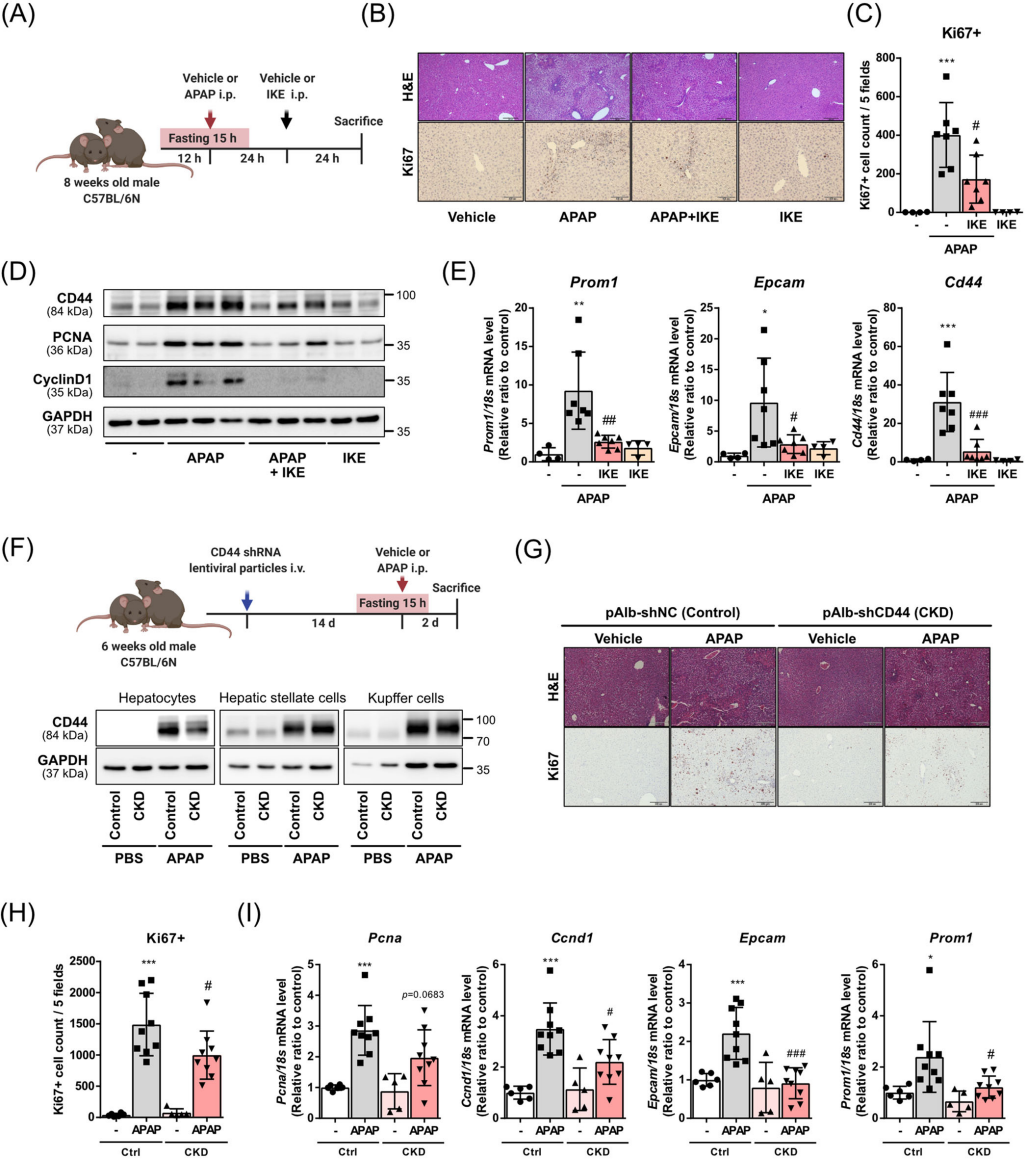
Liver regeneration is abrogated by administration of system x_c_^−^ inhibitor or knockdown of hepatocyte‐specific CD44. (A) Schematic of mice receiving an intraperitoneal injection of vehicle or imidazole ketone erastin (IKE) after APAP dosing. Administration of APAP and IKE were spaced for 24 h. *n* = 4 for PBS‐injected groups, and *n* = 7 for APAP‐injected groups. (B) Representative images of hematoxylin and eosin (H&E) or Ki67 stained liver sections (scale bars, 200 µm or 100 µm). (C) Ki67 positive cells were counted using five fields per Ki67‐stained sample. (D) Immunoblots of CD44, proliferating cell nuclear antigen (PCNA) and CyclinD1 in liver lysates after injection of APAP and IKE. (E) mRNA expression levels of hepatic progenitor cell (HPC) markers (*Cd44, Prom1* and *Epcam*) were measured using qPCR. (C and E) Data are presented as mean ± SD; *p < .05, **p < .01 and ***p < .001 compared to PBS‐injected group; #*p *< .05, ##*p *< .01 and ###*p *< .001 compared to APAP‐injected group, analysed by one‐way ANOVA followed by Tukey's test. (F) Schematic of APAP injury in hepatocyte‐specific CD44 knockdown mice. Mice were intravenously injected with either LV‐pAlb‐shNC (control) or LV‐pAlb‐shCD44 (CKD) to specifically delete CD44 in hepatocytes; i.v, intravenous; i.p., intraperitoneal. 12 h‐fasted control and CKD mice were injected with APAP (250 mg/kg) or PBS 2 weeks after lentiviral particle‐injection. Protein expression of CD44 in isolated primary cells from control or CKD mice after PBS or APAP injection. (G) Representative images of H&E or Ki67 stained liver sections, (scale bars, 200 µm). *n* = 6 for control‐PBS injected group, *n* = 9 for control‐APAP injected group, *n* = 5 for CKD‐PBS injected group and *n* = 9 for CKD‐APAP injected group. (H) Ki67 positive cells were counted using five fields per Ki67‐stained sample. (I) mRNA expression levels of proliferation markers (*Pcna* and *Ccnd1*), and HPC markers (*Epcam* and *Prom1*) were measured in liver tissues using qPCR. (H and I) Data are presented as mean ± SD; *p < .05 and ***p < .001 compared to vehicle group (LV‐pAlb‐shNC i.v. injection followed by PBS i.p. injection); #*p *< .05 and ###*p *< .001 compared to APAP‐treated group (LV‐pAlb‐shNC i.v. injection followed by APAP i.p. injection) analysed by one‐way ANOVA followed by Tukey's test

Despite their small population in the liver, Kupffer cells and hepatic stellate cells highly express CD44^9^, ^10^ (Figure S4E). Therefore, we produced a lentiviral ‐albumin promoter‐shCD44 (LV‐pAlb‐shCD44) delivery system to accomplish hepatocyte‐specific CD44 knockdown (Figure 4F, Figure S4F). CD44 knockdown reduced xCT expression in the plasma membrane of isolated hepatocytes (Figure S4G–J). Moreover, injection of pAlb‐shCD44 significantly inhibited the regenerative process following APAP injection (Figure 4G–I). Likewise, non‐selective CD44 knockdown by U6‐shCD44 delivery decreased the regeneration rate (Figure S5A–D). Taken together, these results confirmed that CD44 increase is essential to support HPC proliferation.

The obtained results delineate the central role of CD44 in modulating liver‐regenerative capacity. Specifically, CD44 contributes to the intracellular redox balance and cell proliferation by enhancing extracellular cystine uptake through system x_c_^−^.

## CONFLICT OF INTEREST

The authors declare no conflict of interest.

## Supporting information

Supporting information
