# Supplementary material for: CD44 is involved in liver regeneration through enhanced uptake of extracellular cystine
Source: Clin Transl Med. 2022 May 11;12(5):e873. doi: 10.1002/ctm2.873 (PMC9091989; doi:10.1002/ctm2.873)
Supplement: Supplementary file 1 — Supporting information [file CTM2-12-e873-s001.docx]

**Supporting Information**

CD44 is involved in liver regeneration through enhanced uptake of extracellular cystine

**Methods and Materials**

**Animals**

C57BL/6 mice aged 6-8 weeks were obtained from Orientbio (Sungnam, Korea). The mice were acclimatized for at least 1 week before use. All mice were housed at a temperature of 21 ± 2°C with 50 ± 5% humidity on a 12 h light/dark cycle. Animal experiments were performed in accordance with protocols approved by the Institutional Animal Care and Use Committee (IACUC; SNU-210628-10 and SNU-210723-1) and the Institutional Biosafety Committee (IBC; SNUIBC-R210405-3) in Seoul National University.

**APAP induced liver injury**

For the acute model of liver injury, mice were fasted for 12 h and then intraperitoneally injected with 250 mg/kg of APAP (Sigma-Aldrich, St Louis, MO, USA). APAP was dissolved in 60°C Dulbecco's phosphate-buffered saline (Biowest, Riverside, MO, USA) and cooled to room temperature before administration. Fasting lasted 3 h after injection.

**Resection of the liver**

8 week-old C57BL/6 mice were anesthetized by intraperitoneal injection of Zoletil^®^50 (30 mg/kg, Virbac Laboratories, Carros, France) and Rompun^®^ (10 mg/kg, Bayer Korea, Korea) mixture. Mice were subjected to hepatectomy of the left lobe with a mean tissue weight of 0.25 ± 0.02 g. The resected volume consisted of approximately 30% of the whole liver, and more than 80% of the left lobe. At the end of the resection, warm normal saline was inserted into the abdominal cavity to replenish fluid loss. The abdomen and skin were closed in layers using running 3/0 silk sutures. Mice were then placed on heat pads until termination of anesthesia. The liver tissues were sampled for further experiments after 1, 4, 7, 10, or 20 days of recovery.

**Isolation of primary hepatocytes**

Primary hepatocytes were isolated from C57BL/6 mice using non-recirculating 2-step perfusion method using calcium and magnesium-free Hanks’ salt solution. Liver tissues from 8-10 week-old mice were perfused with digestion buffer containing 0.5 mM EGTA (Invitrogen, Carlsbad, CA, USA) and 1 mg/ml collagenase (Sigma-Aldrich, St Louis, MO, USA). The cell suspension was filtered through a 70 μm cell strainer and centrifuged at 50 × g for 5 min. Isolated hepatocytes were plated on a collagen-coated plate (Corning, New York, NY, USA).

CD44-expressing hepatocytes were sorted using cell sorter, FACS AriaII (BD Biosciences, San Jose, CA, USA), after incubation of the hepatocytes with APC anti-CD44 antibody. To obtain HPCs that can proliferate on HA-coated dishes, small hepatocytes were isolated from the hepatic cell suspension. Isolated hepatocytes from mice were centrifuged at 50 × g for 1 min after filtration through a 70 μm cell strainer. Then, the supernatants were centrifuged at 50 × g for 5 min. The cell pellets, gently dissociated by pipetting, were seeded in 5 × 10^5^ cells per well of HA-coated plates. The HPCs were cultured in DMEM/F12 medium containing fetal bovine serum, nicotinamide (10 mM), ascorbic acid-2 phosphate (1 mM), insulin-transferrin-selenium, epidermal growth factor (10 ng/ml) and dexamethasone (40 ng/ml).

**Cell culture**

AML12 cells were cultured in Dulbecco’s modified Eagle’s medium/F12 (Gibco, Waltham, MA, USA) supplemented with 10% fetal bovine serum (Gibco, Waltham, MA, USA), 1% penicillin/streptomycin solution (Hyclone, Logan, UT, USA), 1% insulin-transferrin-selenium (Gibco, Waltham, MA, USA), and 40 ng/ml dexamethasone. The cells were kept at 37ºC in a humidified atmosphere consisting of 5% CO_2_.

**Isolation of hepatic stellate cell and Kupffer cell**

Hepatic nonparenchymal cells were obtained from the supernatant of digested liver cell suspension after centrifugation at 50 × g for 5 min. Primary HSCs were isolated through density-gradient centrifugation using percoll (GE Healthcare, Madison, WI, USA) and ficoll paque-plus (GE Healthcare, Madison, WI, USA). The supernatants were centrifuged at 600 × g. The cell fraction was resuspended in a solution with 9 ml ficoll and 1ml percoll. 1 ml PBS was added carefully at the top, followed by centrifugation at 1,400 × g for 15 min. The layer between PBS and the ficoll/percoll gradient solution was acquired for HSC.

Kupffer cells were isolated through density-gradient centrifugation using Optiprep. The nonparenchymal cell fraction was resuspended in 20% Optiprep. 11.5% Optiprep and HBSS were layered on the suspended cells and centrifuged at 1,811 × g for 17 min. Kupffer cells were acquired from the layer between 11.5% Optiprep and 20% Optiprep.

**Stable isotope tracing**

Universal ^13^C_3_-cysteine was oxidized to universal ^13^C_6_-cystine using 30% hydrogen peroxide according to the previous method. Cultured AML12 and attached primary hepatocytes were incubated with cystine-free media containing 0.2 mM ^13^C_6_-cystine or methionine-free media containing 0.2 mM ^34^S-methionine. After collecting culture media, the resident cells were treated with 80% methanol containing 0.1% perchloric acid and internal standards (ISTDs, 2 μM glutamate-^13^C_5_,^15^N,2,3,3,4,4-d_5_ and 2 μM methyl-D9-betaine). Samples were reduced with 0.3 mM Tris-(2-carboxyethyl)-phosphine hydrochloride at room temperature for 30 min to analyze total cysteine and GSH. After centrifugation at 10,000 × g 4°C for 30 min, the aliquot of the supernatant was further diluted with 5-fold in 80% methanol containing 0.1% perchloric acid and ISTDs for determination of GSH.

The sample analysis was conducted by 10 μl injection using an autosampler and separated by AQUASIL C18 column (2.1 x 150 mm, 3 μm, Thermo Fisher Scientific, Waltham, MA, USA) in an oven maintained at 30°C. The analysis was performed using a Shimadzu 20AD-XR HPLC system with an autosampler, column oven and binary pump (Shimadzu, Kyoto, Japan). The mobile phase consisted of deionized water containing 0.1% formic acid (A) and methanol containing 0.15% formic acid (B). A linear gradient of the two solvents was used at 0 min, 30% B, 1.5 min, 30% B, 4 min 40% B, 4.1 min 80% B, 6 min 80% B, 6.1 min 5% B, 10 min 5% B, 10.1 min 30% B and 13 min 30% B for analysis of SAM, SAH, cystathionine, cysteine, GSH and glutamate-^13^C_5_,^15^N,2,3,3,4,4-d_5_ and at 0 min, 80% B, 1.5 min, 80% B, 2 min 60% B, 5 min 40% B, 6 min 80% B and 8.01 min 80% B for analysis of methionine and methyl-D9-betaine. The column was re-equilibrated for 1 min and the flow rate was 0.2 ml/min. LC-MS/MS data were acquired with an SCIEX API 4000 triple quadrupole mass spectrometer (Applied Biosystems, Foster City, CA, USA) equipped with a Turbo V ionization source.

The multiple reaction monitoring (MRM) information used for the analysis is as follows: methionine (150>133), SAM (399>250), SAH (385>134 and 385>136), cystathionine (223>134), cysteine (122>59), GSH (308>179), glutamate-^13^C_5_,^15^N,2,3,3,4,4-d_5_ (159>93) and methyl-D9-betaine (127>66) with positive mode. Acquisition and analysis of data were performed with Analyst^®^ software (ver.1.5.2; Applied Biosystems, Foster City, CA, USA). Details of metabolite detection are shown in Table S1,2. Isotopologues (from M0 to Mn) abundance profiles from stable isotope tracing experiments were corrected for the presence of naturally occurring isotopes and impurities of the tracer substrates with IsoCorrectoR. Absolute concentrations were calculated by multiplying the volume of cell lysate and dividing the total protein amount. For the determination of relative concentrations, the sum of the ratio of each isotope peak area to ISTD peak area was multiplied by the volume of cell lysate and divided into total protein amount.

**Small interfering RNA transfection**

Negative control small interfering RNA (siRNA) and siRNA targeting mouse CD44 gene were purchased from Bioneer (Daejeon, Korea). Knockdown of CD44 was induced using two siRNAs (siCD44 1, 12505-1; siCD44 #2, 12505-3). siRNAs were transfected to AML12 cells using Lipofector-EZ (AptaBio, Yongin, Korea) according to the manufacturer's protocol. AML12 cells were incubated for 36 h in siRNA containing transfection optimized media. 48 h after transfection, the transfected AML12 cells were used for further experiments.

**Knockout of CD44 gene**

CD44-null AML12 cells were established using CRISPR-Cas9 system. Plasmid DNA (U6-gRNA/CMV-Cas9-RFP plasmid targeting the mouse CD44 exon2, U6-gRNA/CMV-Cas9-GFP plasmid for negative control) were purchased from Sigma (Sigma-Aldrich, St Louis, MO, USA). AML12 cells were transfected using Lipofectamine 2000 (Thermo Fisher Scientific, Waltham, MA, USA), followed by sorting of GFP- or RFP-positive cells using FACSAria II cell sorter (BD Biosciences, San Jose, CA, USA). Single-cell clone was isolated and screened for genetic and functional deletion of CD44.

**Determination of ROS**

Intracellular ROS production was measured using CM-H_2_DCFDA. Briefly, AML12 cells were incubated with 10 μM CM-H_2_DCFDA for 30 min after exposure to tertiary-butyl hydrogen peroxide (tBHP). Green fluorescence intensity and cell confluency were determined by IncuCyte^®^ S3 Live-Cell Analysis System (Essenbio science, Inc., Ann Arbor, MI, USA).

**GSH assay**

Intracellular glutathione (GSH + GSSG) concentrations were measured by GSH assay kit (Cayman Chemical, Ann Arbor, MI, USA) following the manufacturer’s instruction. AML12 cells were collected and sonicated in 50 mM MES buffer containing 1 mM EDTA (pH 6-7).

**Lead sulfide assay for H_2_S production**

H_2_S production was measured by lead sulfide assay. 6 × 10^3^ AML12 cells were cultured in growth media supplemented with indicated concentration of cystine and 1 mM pyridoxal 5-phosphate hydrate (Sigma-Aldrich, St Louis, MO, USA). A piece of Whatman filter paper was soaked in 20 mM lead acetate (Sigma-Aldrich, St Louis, MO, USA) and dried overnight. The paper was placed over the wells in 96-well plate and covered by the plate lid with a heavy object on the top. 24 h after incubation of AML12 cells, the formation of lead sulfide was indicated by the brown circles on the filter paper and recorded with a camera.

**Glutamate assay**

The glutamate level in the phenol red free-medium was measured using the Amplex^®^ Red glutamate release assay kit (Thermo Fisher Scientific, Waltham, MA, USA) according to the manufacturer’s instruction. The glutamate concentrations of the medium were calculated in reference to the standard curve, and then normalized using protein level of the cells determined by Bradford assay.

**Immunoblot assay**

Mouse liver tissues or harvested cells were lysed in lysis buffer with protease inhibitor cocktail (Roche Diagnostics, Mannheim, Germany), and phosphatase inhibitors (Sigma-Aldrich, St Louis, MO, USA), followed by centrifugation to obtain clear lysates. Protein concentration of the lysates were measured by Bradford assay and equal amount of protein lysates were separated using sodium dodecyl sulfate-polyacrylamide gel electrophoresis (SDS–PAGE). The fractionated proteins were transferred to nitrocellulose paper (GE healthcare, Madison, WI, USA). The membranes were blocked with 5% skim milk or 5% bovine serum albumin and subjected to immunoblot analysis using the indicated primary antibodies (Table S3). Horseradish peroxidase conjugated IgG antibodies (Cell Signaling Technology, Beverly, MA, USA) were used as the secondary antibodies. Immune complexes were visualized using the Immobilon Western Chemiluminescent HRP Substrate (Merck Millipore, Billerica, MA, USA). Densitometric protein levels were quantified by the image software, Multi Gauge, V3.0 (FUJIFILM, Tokyo, Japan).

**Immunoprecipitation**

AML12 cells were lysed in IP lysis buffer supplemented with phosphatase inhibitors (Sigma-Aldrich, St Louis, MO, USA) and protease inhibitor (Roche Diagnostics, Mannheim, Germany). 500 μg of protein in cell lysis were incubated with 2.5 μg of rabbit polyclonal antibody to CD44 antibody overnight in 4°C. G-agarose beads (Millipore, Bedford, MA, USA) were added and the mixture was incubated for an 2 h on ice. The beads were isolated by centrifugation and washed three times with lysis buffer. The bead-bound proteins were subjected to immunoblot analysis after boiling for 5 min in 95°C.

**Quantitative real-time polymerase chain reaction (qRT-PCR)**

RNA was isolated from mouse tissues and harvested cells using Trizol^®^ reagent (Thermo Fisher Scientific, Waltham, MA, USA). Reverse transcriptase (iNtRON Biotechnology, Seongnam, Korea) and oligo (dT) primers (Table S4) were used to synthesize cDNA. Quantifications of mRNA levels were carried out using SYBR Select Master Mix (Applied Biosystems, Foster City, CA, USA).

**ARE reporter gene assay**

AML12 cells were transfected with ARE reporter gene vector by using lipofectamine-EZ (AptaBio, Yongin, Korea) in accordance with the manufacturer’s instructions. 24 h after transfection, luciferase activity was assessed using the Dual-Luciferase Reporter Assay System (Promega, Madison, CA, USA) and a luminometer (Berthold Technologies, Bad Wildbad, Germany). ARE-promoter-driven firefly luciferase activity was normalized to Renilla luciferase activity.

**Confocal microscopy**

Liver tissues were sectioned at -20°C and left to dry for 1 h. The liver sections were fixed in cold acetone for 15 min prior to brief PBS washes, permeabilized with 0.1% TritonX-100 (Sigma-Aldrich, St Louis, MO, USA), and blocked with 2.5% normal goat serum for 1 h. The sections were incubated with PCNA and CD44 primary antibodies overnight in 4°C, followed by incubation with the appropriate secondary antibodies, Alexa Fluor 488 or Alexa Fluor 594 (Thermo Fisher Scientific, Waltham, MA, USA).

Cultured cells were fixed with 4% paraformaldehyde solution for 15 min, followed by permeabilization using 0.1% TritonX-100. The fixed cells were blocked with horse serum for 1 h and incubated with CD44 and 4F2hc primary antibodies overnight at 4°C. After thorough PBS washing, the cells were incubated with Alexa Fluor 488 or Alexa Fluor 568 conjugated-secondary antibodies. The fluorescence was detected using confocal microscope, Leica TCS SP8 MP (Leica, Wetzlar, Germany). For colocalization analysis, Pearson’s correlation coefficient was calculated using Image J software (National Institute of Health, Bethesda, MD, USA). The comparison was performed on 9 sets of fluorescent images acquired with the same optical settings.

**Assessment of liver injury**

Liver tissues were fixed in 10% neutral-buffered formalin solution (Sigma-Aldrich, St Louis, MO, USA), embedded in paraffin, and stained for histology. Photographs of stained liver sections were taken at a magnification of ×100. Ki67 stained nuclei in liver tissues were counted using Image J software (National Institute of Health, Bethesda, MD, USA).

**Lentivirus injection**

The mice were divided into 4 groups (n = 10); Vehicle group (LV-GFP-pAlb-shNC i.v. injection followed by PBS i.p. injection), APAP group (LV-GFP- pAlb-shNC i.v. injection followed by APAP i.p. injection), pAlb-shCD44 group (LV-GFP-pAlb-shCD44 i.v. injection followed by PBS i.p. injection) and pAlp-shCD44 + APAP group (LV-GFP-pAlb-shCD44 i.v. injection followed by APAP i.p. injection). We used Lenti-shCD44 (shCD44 Lenti-Virus) to knockdown CD44. 6-week-old mice were tail-vein injected with Lenti-Virus (2 × 10^7^ PFU/mouse). The lentivirus was obtained from Pharos Vaccine (Seongnam, Korea). The lentivirus targeted 21 nucleotides of mouse CD44, GGTAATTCCGAGGATTCATCC. 2 weeks after lentivirus injection, mice were challenged with APAP injection and sacrificed.

**Statistical analysis**

Data are mean ± SD. Statistical significance between two groups was assessed by student’s *t*-test. Comparisons of multiple groups were evaluated using one-way ANOVA, followed by Tukey’s test or Dunnett’s test. *p*-value < 0.05 was considered statistically significant. GraphPad PRISM software (GraphPad Software Inc., San Diego, CA, USA) was used for statistical analysis.

**Supplementary Tables**

| **Table S1. Isotope pattern analysis for ^13^C_6_-cystine metabolic tracing** | | | | | | | |
| --- | --- | --- | --- | --- | --- | --- | --- |
| Metabolites | Formula | Isotopologue | MRM (m/z) | Product ion Formula | DP | CE | CXP |
| Cysteine | C3H7N1O2S1 | M0>m0 | 122>59 | [C2H3S1]+ | 41 | 29 | 10 |
|  |  | M1>m0 | 123>59 |  | 41 | 29 | 10 |
|  |  | M1>m1 | 123>60 |  | 41 | 29 | 10 |
|  |  | M2>m1 | 124>60 |  | 41 | 29 | 10 |
|  |  | M2>m2 | 124>61 |  | 41 | 29 | 10 |
|  |  | M3>m2 | 125>61 |  | 41 | 29 | 10 |
|  |  |  |  |  |  |  |  |
| GSH | C10H17N3O6S1 | M0>m0 | 308>179 | [C5H11N2O3S1]+ | 59 | 17 | 9 |
|  |  | M1>m0 | 309>179 |  | 59 | 17 | 9 |
|  |  | M1>m1 | 309>180 |  | 59 | 17 | 9 |
|  |  | M2>m0 | 310>179 |  | 59 | 17 | 9 |
|  |  | M2>m1 | 310>180 |  | 59 | 17 | 9 |
|  |  | M2>m2 | 310>181 |  | 59 | 17 | 9 |
|  |  | M3>m0 | 311>179 |  | 59 | 17 | 9 |
|  |  | M3>m1 | 311>180 |  | 59 | 17 | 9 |
|  |  | M3>m2 | 311>181 |  | 59 | 17 | 9 |
|  |  | M3>m3 | 311>182 |  | 59 | 17 | 9 |

| **Table S2. Isotope pattern analysis for ^34^S-methionine metabolic tracing** | | | | | | | | |
| --- | --- | --- | --- | --- | --- | --- | --- | --- |
| Metabolites | | Formula | Isotopologue | MRM (m/z) | Product ion Formula | DP | CE | CXP |
| Methionine | C5H11N1O2S1 | | M0>m0 | 150>133 | [C5H9O2S1]+ | 41 | 15 | 18 |
|  |  | | M2>m2 | 152>135 |  | 41 | 15 | 18 |
|  |  | |  |  |  |  |  |  |
| SAM | C15H22N6O5S1 | | M0>m0 | 399>250 | [C10H12N5O3]+ | 66 | 21 | 14 |
|  |  | | M2>m0 | 401>250 |  | 66 | 21 | 14 |
|  |  | |  |  |  |  |  |  |
| SAH | C14H20N6O5S1 | | M0>m0 | 385>134 | [C4H8NO2S1]+ | 60 | 24 | 11 |
|  |  | | M0>m0 | 385>136 | [C5H6N5]+ | 60 | 24 | 11 |
|  |  | | M2>m0 and M2>m2 | 387>136 |  | 60 | 24 | 11 |
|  |  | |  |  |  |  |  |  |
| Cystathionine | C7H14N2O4S1 | | M0>m0 | 223>134 | [C4H8N1O2S1]+ | 46 | 20 | 7 |
|  |  | | M2>m2 | 225>136 |  | 46 | 20 | 7 |
|  |  | |  |  |  |  |  |  |
| Cysteine | C3H7N1O2S1 | | M0>m0 | 122>59 | [C2H3S1]+ | 41 | 29 | 10 |
|  |  | | M2>m2 | 124>61 |  | 41 | 29 | 10 |
|  |  | |  |  |  |  |  |  |
| GSH | C10H17N3O6S1 | | M0>m0 | 308>179 | [C5H11N2O3S1]+ | 59 | 17 | 9 |
|  |  | | M2>m2 | 310>181 |  | 59 | 17 | 9 |

**Table S3. Antibody List**

| Target | Supplier | Cat.no | Use |
| --- | --- | --- | --- |
| CD44 | Abcam | ab157107 | WB, IF, IP |
| CD44  (APC anti-CD44) | Biolegend | 103012 | Flow cytometry |
| PCNA | Santa-cruz | sc-56 | WB |
| GAPDH | Millipore | CB1001 | WB |
| β-actin | Sigma | a2228 | WB |
| 4F2hc | Santa-cruz | sc-390154 | WB |
| xCT | Novus | NB300-318 | WB |
| Ecadherin | BD science | 610181 | WB |
| CyclinD1 | Cell Signaling | 2978s | WB |
| Albumin | Cell Signaling | 4929s | WB |
| CSE | Abnova | H00001491-M01 | WB |
| CBS | Santa-cruz | sc-133154 | WB |
| Nrf2 | Santa-cruz | sc-722 | WB |
| Rabbit normal IgG | Abcam | ab37415 | IP |

**Table S4. Primer List**

| Name | Sequence | Supplier |
| --- | --- | --- |
| *Cd44* primer for qPCR | Forward (5'→3')  ACAGTACCTTACCCACCATG  Reverse (5'→3')  GGATGAATCCTCGGAATT | Bioneer |
| *Slc7a11* primer for qPCR | Forward (5'→3')  CCTGGCATTTGGACGCTACAT  Reverse (5'→3')  TCAGAATTGCTGTGAGCTTGCA | Bioneer |
| *Slc3a2* primer for qPCR | Forward (5'→3')  CTCCCAGGAAGATTTTAAAGACCTTCT  Reverse (5'→3')  TTCATTTTGGTGGCTACAATGTCAG | Bioneer |
| *Ccnd1* primer for qPCR | Forward (5'→3')  GCGTACCCTGACACCAATCTC  Reverse (5'→3')  CTCCTCTTCGCACTTCTGCTC | Bioneer |
| *Pcna* primer for qPCR | Forward (5'→3')  GCTTGGCAATGGGAACATT  Reverse (5'→3')  TCATCTTCAATCTTGGGAGC | Bioneer |
| *Prom1* primer for qPCR | Forward (5'→3')  CTCCCATCAGTGGATAGAGAACT  Reverse (5'→3')  ATACCCCCTTTTGACGAGGCT | Bioneer |
| *Epcam* primer for qPCR | Forward (5'→3')  AGAATACTGTCATTTGCTCCAAACT  Reverse (5'→3')  GTTCTGGATCGCCCCTTC | Bioneer |
| *Ho-1* primer for qPCR | Forward (5'→3')  TTACCTTCCCGAACATCGAC  Reverse (5'→3')  GCATAAATTCCCACTGCCAC | Bioneer |
| *Nqo1* primer for qPCR | Forward (5'→3')  GGCATCCTGCGTTTCTGTG  Reverse (5'→3')  GGTTTCCAGACGTTTCTTCCAT | Bioneer |
| *Gclc* primer for qPCR | Forward (5'→3')  AATGGAGGCGATGTTCTTGAG  Reverse (5'→3')  CAGAGGGTCGGATGGTTG | Bioneer |
| *Gclm* primer for qPCR | Forward (5'→3')  GGCTTCGCCTCCGATTGAAGA  Reverse (5'→3')  TCACACAGCAGGAGGCCAGGT | Bioneer |
| *Gst2a* primer for qPCR | Forward (5'→3')  CTATGTTGAAGAGCTTGATGCC  Reverse (5'→3')  ACTTGAAAACCTTCCTTGCTTC | Bioneer |
| *Gpx2* primer for qPCR | Forward (5'→3')  ACCGATCCCAAGCTCATCAT  Reverse (5'→3')  CAAAGTTCCAGGACACGTCTGA | Bioneer |

**Supplementary Figures**


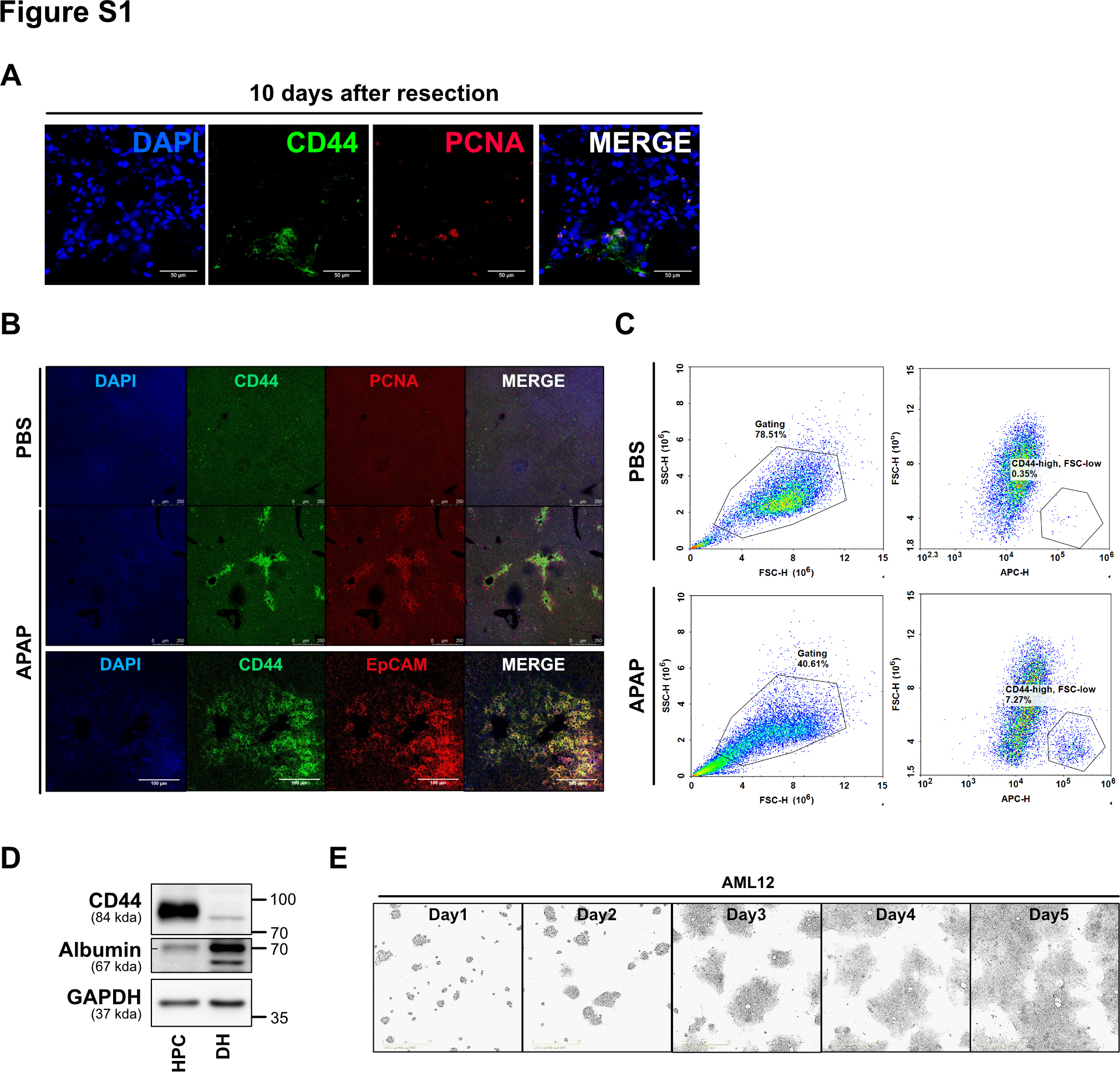


**Figure S1. Distributions of CD44-APC stained primary hepatocytes**

(A) Immunofluorescence images (Scale bars, 50 μm) of CD44 and PCNA in liver tissues from mice 10 days after resection. (B) Immunofluorescence images (Scale bars, 250 μm) of CD44 and PCNA in liver tissues from mice 48 h after PBS or APAP injection. Immunofluorescence images of CD44 and EpCAM in liver tissues from mouse 48 h after APAP injection (Scale bars, 100 μm). (C) FSC and SSC were measured in CD44-APC stained primary hepatocytes using flow cytometry. Hepatocytes from PBS or APAP-injected mice were isolated, and a total of 10,000 live cells were gated using FSC and SSC. FSC of CD44-high hepatocytes were analyzed among the gated populations. This experiment is the representative of three different samples; APC, allophycocyanin; FSC, forward scatter; SSC, side scatter. (D) Small hepatocytes cultured in an HA-coated dish for 14 days were considered as hepatic progenitor cells (HPC). Mouse primary hepatocytes were indicated as differentiated hepatocytes (DH). Protein levels of albumin and CD44 were measured in HPC and DH. (E) Representative images (Scale bars, 400 μm) of AML12 cells cultured on an HA-coated dish.


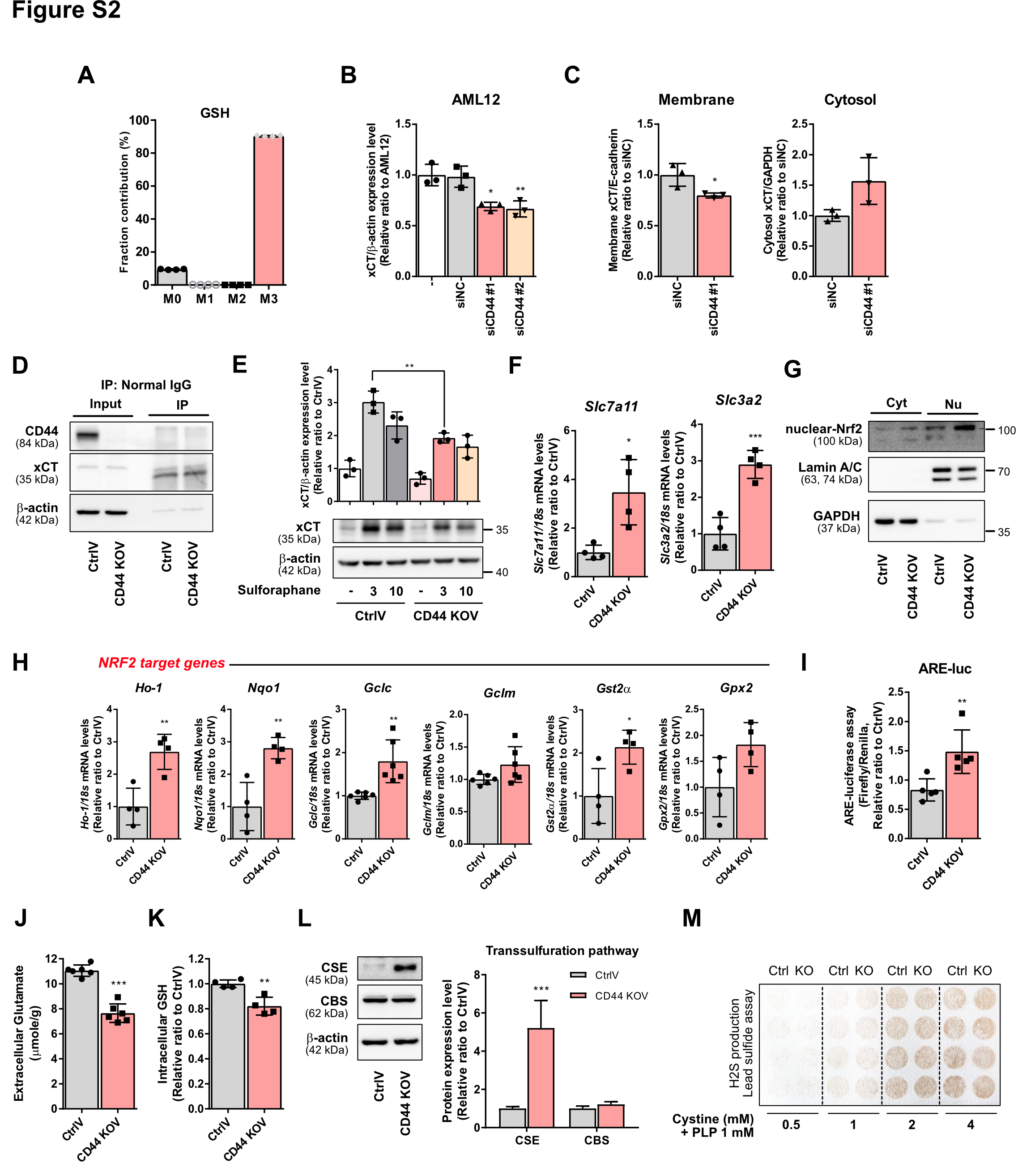


**Figure S2. Silencing of CD44 using siCD44 or CRISPR-Cas9 system**

(A) AML12-CtrlV was incubated in media containing universal ^13^C_6_-cystine. ^13^C-labeled GSH was measured in lysates of AML12, 12 h after the medium change. The bar graph visualizes the proportion of GSH that contains the stable isotope. (B) Protein expression of xCT was measured in CD44-knockdown AML12. (C) AML12 cells transfected with CD44 siRNA were fractionated to obtain plasma membrane and cytosol samples and then subjected to immunoblot analysis. Cyt; Cytosolic fraction, Mem; membrane fraction. (D) Lysates of Crispr-sgCtrl (CtrlV) and Crispr-sgCD44 (CD44 KOV) cells were subjected to immunoprecipitation using normal rabbit IgG antibody. (E) AML12 cells were incubated with sulforaphane for 12 h. Protein expression of xCT and β-actin were measured in cell lysates after the incubation. n=3 per group; data are presented as mean ± SD, ***p* < 0.01 compared to an indicated group, analyzed by one-way ANOVA followed by Tukey’s test. (F) Basal mRNA expression of xCT (*Slc7a11*) *a*nd 4F2hc (*Slc3a2*) was quantified using qPCR. n=4 per group. (G) Immunoblot analysis of the nuclear and cytoplasmic fractions of AML12 cells expressing sgCtrl or sgCD44. Cyt; Cytosolic fraction, Nu; nuclear fraction. (H) mRNA levels of NRF2 target genes were measured in AML12-CtrlV, and AML12-CD44 KOV cells. n=4 for each group. (I) Regulation of the NRF2/ARE pathway was determined by ARE-luciferase (ARE-luc) assay. n=5 per group. (J) Extracellular glutamate was measured in the media after incubation of AML12 CtrlV or CD44 KOV cells for 5 h. n=6 per group. (K) Intracellular GSH level was measured using the GSH assay kit. Lysates of AML12 CtrlV or CD44 KOV cells were obtained by sonication. n=4 per group. (L) Protein expression levels of transsulfuration enzymes were quantified as relative densitometric ratio. n=4 for each group; cystathionine β synthase (CBS) and cystathionine γ lyase (CSE). (M) The production of H_2_S by AML12 cells was analyzed 24 h after incubation with the indicated concentration of cystine and pyridoxal phosphate (PLP) using the lead sulfide assay (n = 3 replicates per group). (B, C, F, and H-L) Data are presented as mean ± SD; ****p* < 0.001, ***p* < 0.01, and **p* < 0.05 compared to siNC or CtrlV group, analyzed by unpaired student’s *t*-test.


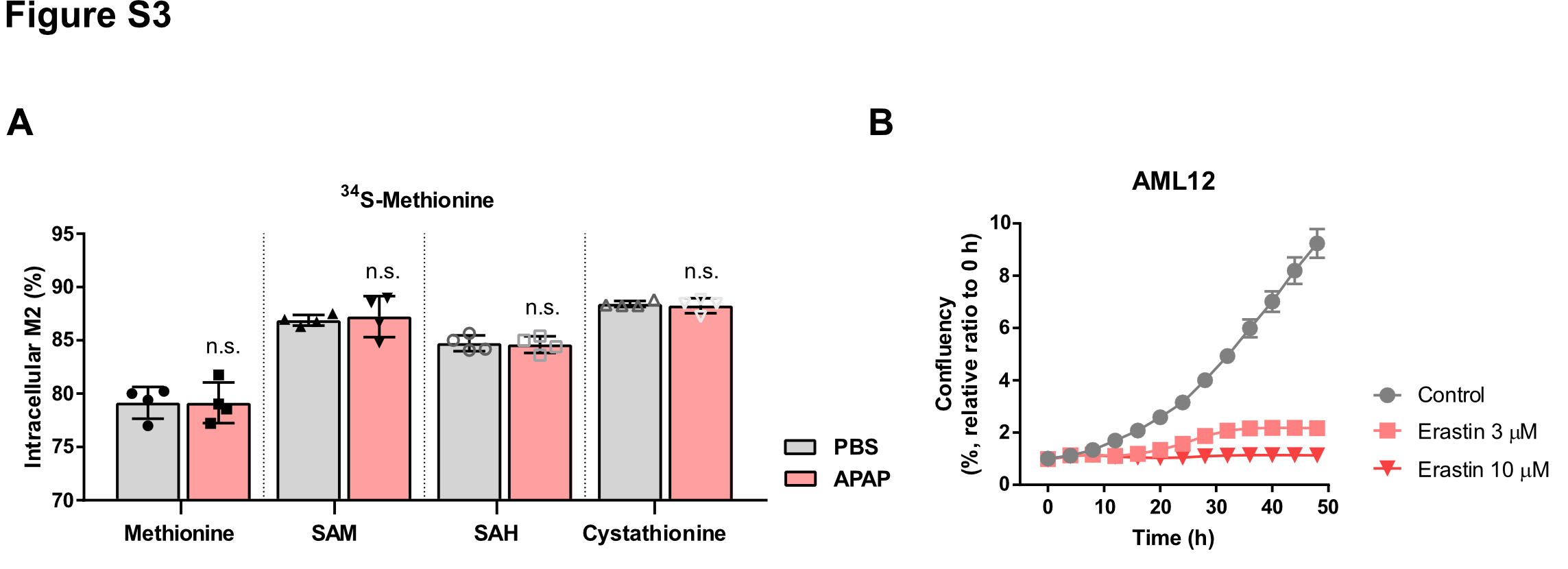


**Figure S3.** **^34^S-methionine isotope tracing and the effect of erastin on AML12 proliferation**

(A) Primary hepatocytes were incubated with methionine-free medium supplemented with 0.2 mM ^34^S-methionine. Methionine, S-adenosylmethionine (SAM), S-adenosylhomocysteine (SAH), and cystathionine were measured in cell lysates after 6 h incubation. n=4 for each group; n.s., not significant. (B) The effects of erastin on cell proliferation of AML12 were assessed using IncuCyte^®^ S3.


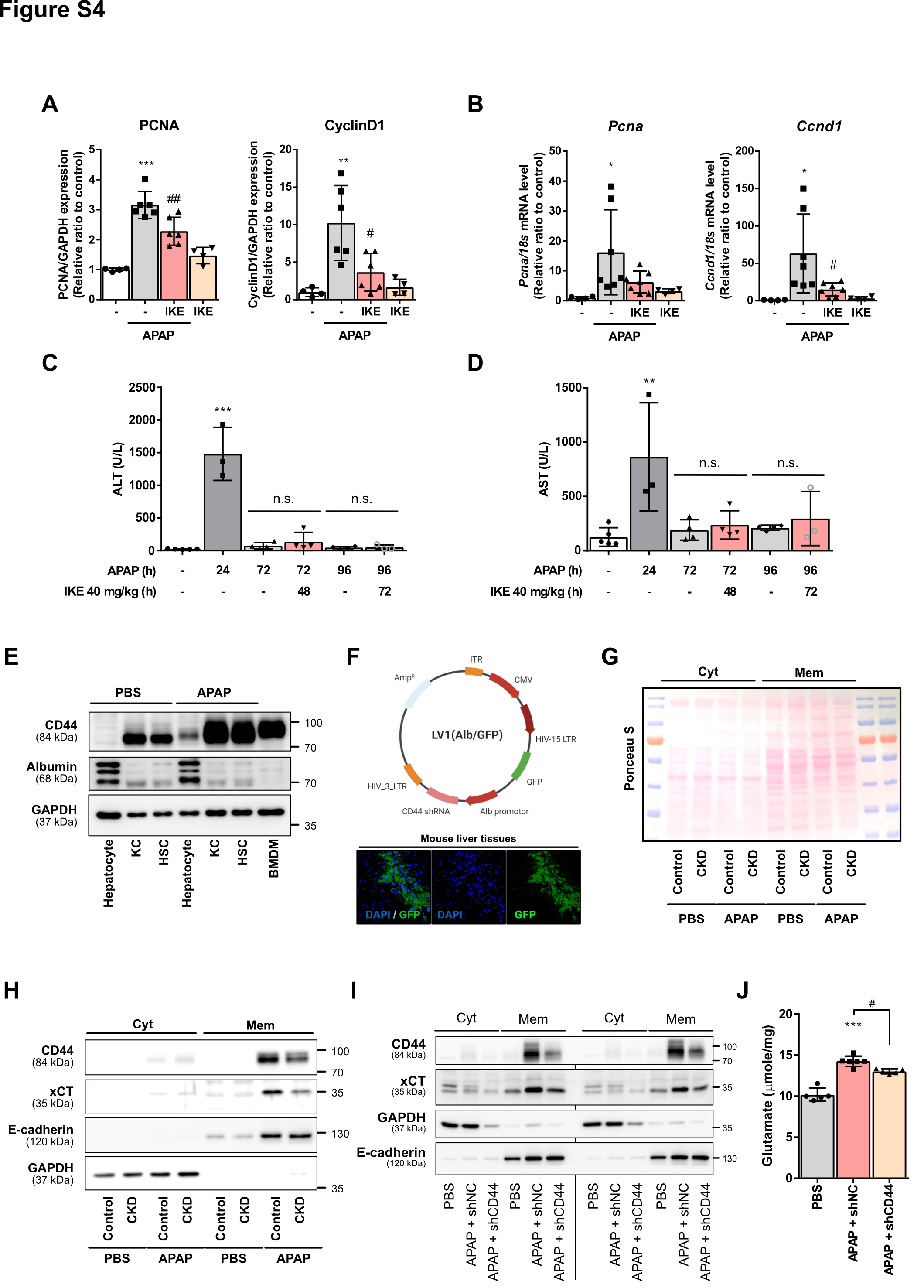


**Figure S4. The effect of system x_c_*^-^* inhibitor or hepatocyte-specific CD44 knockdown on liver regeneration**

(A) C57BL/6 mice were administered imidazole ketone erastin (IKE) following APAP injection, and then sacrificed. Protein expression levels of proliferation markers, PCNA and CyclinD1, were quantified using densitometric analyses. (B) mRNA expression levels of *Pcna* and *Ccnd1* were measured using qPCR. (A and B) Data are presented as mean ± SD; **p* < 0.05, ***p* < 0.01, and ****p* < 0.001 compared to PBS-injected group; #*p* < 0.05, and ##*p* < 0.01 compared to APAP-injected group, analyzed by one-way ANOVA followed by Tukey’s test. (C and D) Serum aspartate aminotransferase (AST) and alanine aminotransferase (ALT) were measured in a time course. Data are presented as mean ± SD; ***p* < 0.01 and ****p* < 0.001 compared to control group; n.s. not significant compared to indicated group, analyzed by unpaired student’s t-test. (E) CD44 expression was assessed in isolated primary cells from PBS or APAP-injected mice; hepatocyte, Kupffer cell (KC), and hepatic stellate cell (HSC). Bone marrow cells were isolated from the tibia and femur of mice. Using macrophage colony-stimulating factor, bone marrow cells were differentiated (7 days) into bone marrow-derived macrophages (BMDMs). (F) Map of LV-pAlb-shCD44 (CKD) vector used to specifically delete CD44 in hepatocytes. (G and H) Hepatocytes from control or CKD mice were fractioned to obtain plasma membranes and cytosol samples and both the fraction samples were subjected to immunoblot analysis. Cyt; cytosolic fraction, Mem; membrane fraction. Ponceau S staining of the same blot is shown as a loading control. (I) CD44 was knocked down in primary hepatocytes isolated from APAP-injured mice using U6-shCD44 lentivirus. The expression of xCT was measured using western blot analysis. (J) Glutamate levels of the supernatants were quantified to evaluate system x_c_^-^ activity. Data are presented as mean ± SD; ****p* < 0.001 compared to PBS-injected group; #*p* < 0.05 compared to indicated group, analyzed by one-way ANOVA followed by Tukey’s test.


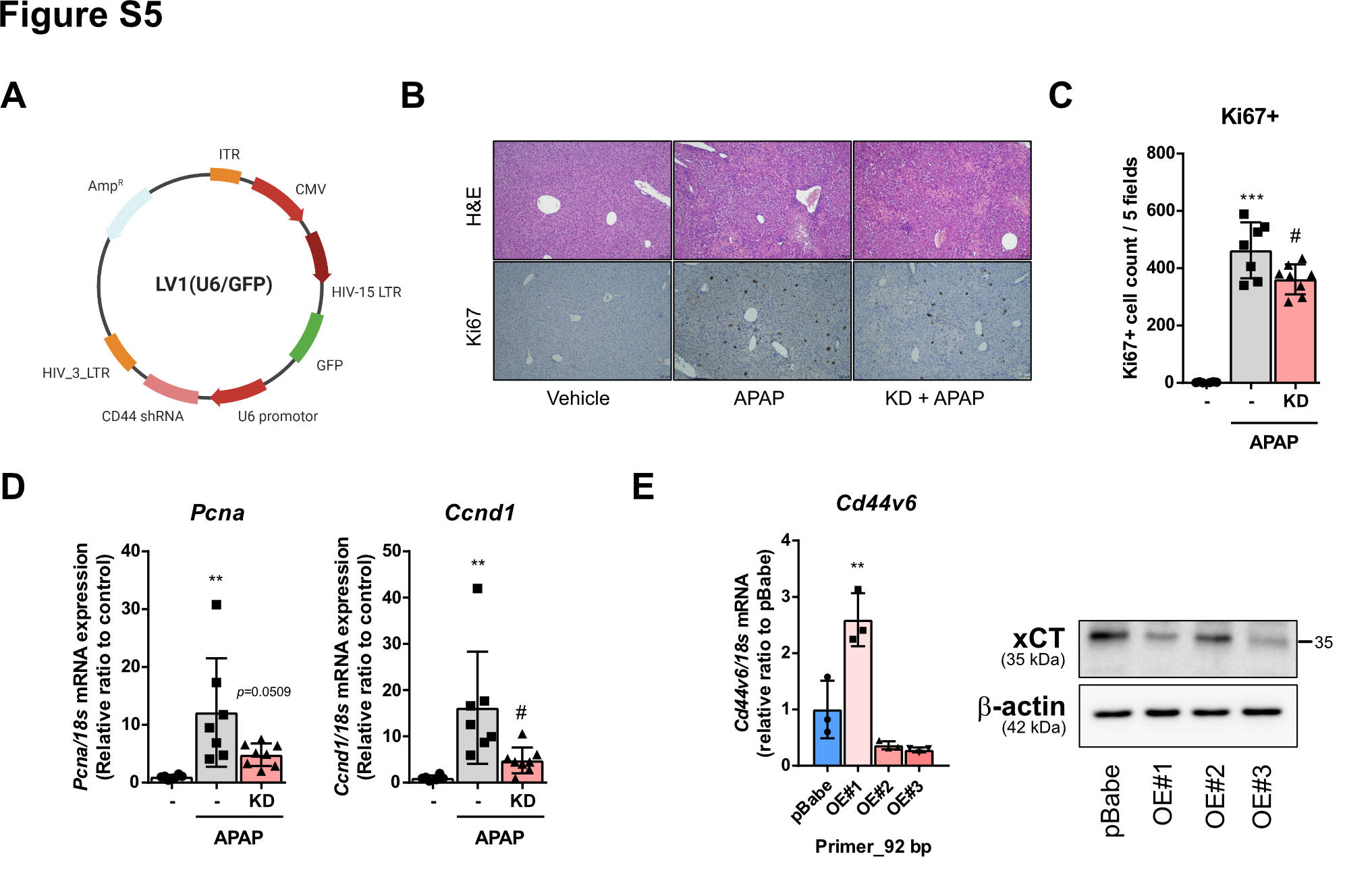


**Figure S5. Knockdown of CD44 *in vivo* using lentiviral injection**

(A) Mice were intravenously injected with LV-U6-shCD44 (KD) to delete CD44. 12 hour-fasted knockdown mice were injected with APAP (250 mg/kg) or PBS 2 weeks after virus injection n=6 for PBS-injected groups, n=7 for APAP-injected group, and n=8 for KD-APAP injected group. (B) Representative images of H&E or Ki67 stained liver sections, (Scale bars, 100 μm). (C) Ki67 positive cells were counted using 5 fields per Ki67-stained sample. (D) mRNA expression levels of proliferation markers (*Pcna* and *Ccnd1*). Data are presented as mean ± SD; ***p* < 0.01, versus control group; #*p* < 0.05 versus APAP-injected group by one-way ANOVA followed by Tuckey’s test. (E) Mouse CD44v6 was overexpressed in AML12 cells using the retroviral vector, pBabe-CD44v6 (Addgene, 107970). We obtained three single-cell colonies (OE#1, OE#2, and OE#3) and compared them to AML12 cells transfected with an empty retroviral vector (Addgene, 10668).

**Discussion**

In this study, we present evidence that the increase of CD44 after fulminant liver injuries promotes liver regeneration by enhancing system x_c_*^-^*-mediated uptake of extracellular cystine. This metabolic change was achieved by stabilizing xCT through the binding of CD44 in the plasma membrane. Knockdown of CD44 in AML12 revealed that CD44 regulates the expression of xCT. CD44-deletion impaired the protein stability of xCT, thus damping the activity of glutamate-cystine exchange. Moreover, the expression of xCT in the plasma membrane was in parallel with the increase of CD44 by APAP-induced liver regeneration. In primary hepatocytes from APAP-injured liver, CD44-dependent xCT regulation resulted in great dependency on extracellular cystine for maintenance of intracellular cysteine and GSH. Administration of IKE or lentiviral delivery of shCD44 inhibited cystine uptake, thereby delaying liver regeneration after APAP injection.

Despite decades of research, the therapeutic options for APAP poisoning are extremely limited. N-acetyl-cysteine is the only clinically proven pharmacologic intervention for APAP overdose within 8 h of APAP ingestion^1^. Whereas liver injury is difficult to manipulate as it often occurs before most patients seek medical attention, liver regeneration can be modulated even in the late stages of APAP-induced liver failure^2^. Therefore, it is apparent that a therapeutic strategy to promote compensatory liver regeneration following APAP overdose is a crucial determinant of outcomes. Here, we suggest a plausible therapeutic approach to enhance liver regeneration. Because CD44 promotes ROS defense and cell proliferation through interaction with system x_c_*^-^*, CD44-targeted delivery of xCT inducer may enhance the liver’s self-renewal capabilities. A recent study reported that pharmacological activation of Nrf2 enhances functional liver regeneration^3^. Based on the notion that Nrf2 upregulates xCT expression, we can posit that CD44-targeted delivery of Nrf2 inducers may enhance the regeneration process that follows APAP overdose.

In the present study, we investigated the actions of CD44 on cysteine metabolism in HPCs and unpacked its role in liver regeneration. HPC-mediated liver regeneration was induced by resection of the hepatic left lobe or APAP exposure. CD44 was commonly elevated in hepatocytes after both liver injuries, but this increased CD44 expression was not sustained in the regenerating livers and ultimately diminished. Given that CD44 is a well-known cancer stem cell marker, its genetic manipulation may affect hepatocyte carcinogenesis. However, our data supports the hypothesis that CD44 increase after such severe liver damage is not a marker for cancer progenitor cells but rather a cell surface marker that is involved in the liver regeneration process.

CD44 is a transmembrane glycoprotein that includes several isoforms with different extracellular regions. It is reported that the CD44 variant isoforms (CD44v), especially CD44v8-10 (isoforms that contain the sequences encoded by variant exons 8-10), interact with xCT at the plasma membrane of cancer cells^4^. In this study, however, we knocked down all CD44 isoforms from CD44v to the standard isoform of CD44 (CD44s) using siRNA, CRISPR-Cas9, or lentiviral shRNA. We could not specify the variant isoforms that mainly regulate system x_c_^-^. Based on the previous report, the expression of CD44 and its isoform, CD44v6, increases in the rat regenerating liver^5^. Besides, we found that the overexpression of CD44v6 in AML12 cells does not increase xCT protein expression (Figure S5E). The variant isoform that regulates system x_c_^-^ needs to be further elucidated.

As an adhesion molecule, CD44’s role has been widely studied in immune cell infiltration and wound-healing processes^6,7^. The investigations into hepatic CD44 have been focused on HSCs and Kupffer cells and in contexts where CD44 is distributed mainly in normal liver tissue. Therefore, previous studies on CD44 in metabolic liver diseases have focused on its contribution to nonparenchymal cells. Patouraux *et al.* demonstrated that CD44 enhances NASH progression by facilitating infiltration of hepatic macrophages and M1 polarization^8^. Moreover, Höchst *et al.* revealed that CD44 in activated HSCs induces myeloid-derived suppressor cells from peripheral blood monocytes and regulates immune functions in the liver^9^. Unlike these previous studies, we investigated the novel role of CD44 in the restoration of liver parenchyma. CD44 is not expressed in normal parenchymal cells such as hepatocytes and cholangiocytes. Following severe liver injury, however, CD44-expressing HPCs proliferate to achieve functional liver reconstitution^10^. Our data revealed that about 8% of hepatocytes expressed high levels of CD44. Considering that hepatocytes constitute a major cellular constituent of the liver, the subpopulation of CD44-expressing HPCs can be regarded as key hepatic cells that contribute to liver regeneration.

The proliferation of HPCs is essential to the self-renewal capacities of the liver. However, another major concern regarding promotion of HPC proliferation is the possibility of intervention by nonparenchymal cells^11^. During and after liver injuries, quiescent HSCs are activated to myofibroblast-like cells that drive liver fibrosis. Because HPCs reside in niches including HSCs and Kupffer cells, a therapeutic strategy to selectively support HPC proliferation while suppressing macrophage infiltration and HSC activation is ideal for recovery from severe liver damage^12,13^. Therefore, the effect of CD44 on cell types other than hepatocytes in liver regeneration should be further dissected. In a previous study, the neutralization of CD44 using a specific antibody partially abrogated hepatic inflammation and macrophage recruitment induced by methionine- and choline-deficient diet (MCDD) challenge^8^. Blockade of CD44 ameliorated liver fibrosis induced by partial inferior vena cava ligation^14^. However, based on our data regarding hepatocyte-specific CD44 deletion, CD44 in HPCs may be critical to the regeneration process following liver injury. Likewise, in this study, non-selective CD44 knockdown by U6-shCD44 delivery decreased the regeneration rate. We postulate that this discrepancy originates from the difference in the HPC contribution to liver restoration. In fulminant liver injury which HPC is critical for its recovery, lack of HPC proliferation delays liver regeneration regardless of the role of CD44 in nonparenchymal cells^7,15^. The importance of nonparenchymal cells activation and HPC proliferation in disease progression should be thoroughly investigated to fully assess the role of CD44 after liver injury.

**Reference**

1. Widjaja AA, Dong J, Adami E, et al. Redefining IL11 as a regeneration-limiting hepatotoxin and therapeutic target in acetaminophen-induced liver injury. *Science translational medicine*. 2021;13(597)

2. Bhushan B, Apte UJTAjop. Liver regeneration after acetaminophen hepatotoxicity: mechanisms and therapeutic opportunities. 2019;189(4):719-729.

3. Chan BK, Elmasry M, Forootan SS, et al. Pharmacological Activation of Nrf2 Enhances Functional Liver Regeneration. *Hepatology*. 2021;

4. Nagano O, Okazaki S, Saya H. Redox regulation in stem-like cancer cells by CD44 variant isoforms. *Oncogene*. 2013;32(44):5191-5198.

5. Della Fazia MA, Pettirossi V, Ayroldi E, Riccardi C, Magni MV, Servillo G. Differential expression of CD44 isoforms during liver regeneration in rats. *Journal of hepatology*. 2001;34(4):555-561.

6. Kimura K, Nagaki M, Kakimi K, et al. Critical role of CD44 in hepatotoxin-mediated liver injury. *Journal of hepatology*. 2008;48(6):952-961.

7. Kikuchi S, Griffin CT, Wang S-S, Bissell DM. Role of CD44 in epithelial wound repair: migration of rat hepatic stellate cells utilizes hyaluronic acid and CD44v6. *Journal of Biological Chemistry*. 2005;280(15):15398-15404.

8. Patouraux S, Rousseau D, Bonnafous S, et al. CD44 is a key player in non-alcoholic steatohepatitis. *Journal of hepatology*. 2017;67(2):328-338.

9. Höchst B, Schildberg FA, Sauerborn P, et al. Activated human hepatic stellate cells induce myeloid derived suppressor cells from peripheral blood monocytes in a CD44-dependent fashion. *Journal of hepatology*. 2013;59(3):528-535.

10. Lu W-Y, Bird TG, Boulter L, et al. Hepatic progenitor cells of biliary origin with liver repopulation capacity. *Nature cell biology*. 2015;17(8):971-983.

11. Williams MJ, Clouston AD, Forbes SJ. Links between hepatic fibrosis, ductular reaction, and progenitor cell expansion. *Gastroenterology*. 2014;146(2):349-356.

12. So J, Kim A, Lee S-H, Shin D. Liver progenitor cell-driven liver regeneration. *Experimental & Molecular Medicine*. 2020;52(8):1230-1238.

13. Kitade M, Kaji K, Nishimura N, et al. Blocking development of liver fibrosis augments hepatic progenitor cell‐derived liver regeneration in a mouse chronic liver injury model. 2019;49(9):1034-1045.

14. Osawa Y, Kawai H, Tsunoda T, et al. Cluster of Differentiation 44 Promotes Liver Fibrosis and Serves as a Biomarker in Congestive Hepatopathy. *Hepatology Communications*. 2021;

15. Bria A, Marda J, Zhou J, et al. Hepatic progenitor cell activation in liver repair. 2017;1(2):81-87.
